# Oesophageal atresia with tracheo-oesophageal fistula in a preterm neonate in Limbe, Cameroon: case report & brief literature review

**DOI:** 10.1186/1756-0500-7-692

**Published:** 2014-10-07

**Authors:** Leopold N Aminde, Veronica N Ebenye, Walters T Arrey, Noah F Takah, George Awungafac

**Affiliations:** Department of Paediatrics, Regional Hospital Limbe, Limbe, Cameroon; Faculty of Health Sciences, University of Buea, Buea, Cameroon

**Keywords:** Oesophageal atresia, Trachea-oesophageal fistula, Diagnosis, Preterm, Cameroon

## Abstract

**Background:**

Oesophageal atresia is a congenital anomaly in which there is interruption of the oesophageal lumen resulting in an upper and lower segment. We present the case of a rare sub-type of Oesophageal atresia with proximal trachea-oesophageal fistula associated with Meconium Aspiration Syndrome. This is the first case reported in literature in the South West Region of Cameroon.

**Case presentation:**

A 2 day old preterm male baby who presented as an emergency with difficulty breathing, fever and refusal to feed. Initially managed as early onset neonatal sepsis from meconium aspiration syndrome in which a diagnosis of oesophageal atresia was finally made.

**Conclusion:**

A high index of suspicion for Oesophageal atresia/trachea-oesophageal fistula should prevail when faced with a neonate with the triad: respiratory distress during feeds, regurgitation and persistent frothy salivation. The case discusses the diagnostic dilemma and management difficulties in a preterm neonate with the above association in a low income setting.

## Background

Congenital malformations and their associations are not so rare and contribute to neonatal and infant morbidity and mortality [1]. Their prevalence ranges from 1% to over 4% depending on the place and population studied [2, 3]. We report the case of Oesophageal atresia (OA) with proximal trachea-oesophageal fistula (TOF) associated with Meconium Aspiration Syndrome (MAS) in a preterm infant. Though, a not so common finding, this re- iterates the importance of thorough clinical examination of new borns, especially preterm, keeping in mind congenital abnormalities. This seems to be the first reported in literature from the South West Region of Cameroon.

## Case presentation

A 2 day old preterm (30 weeks) male baby was admitted as an emergency due to difficulty in breathing, fever and refusal to feed. Mother had 3 antenatal visits (no ultrasound scan), no illness during pregnancy and denies any fever during labour. Past history significant for prolonged labour with excess amniotic fluid (meconium stained) at delivery of male preterm of weight 1.9 kg and APGAR 8, 9 at 1 and 5 minutes respectively. On examination, the child was in respiratory distress (RR = 65 cycles/min) febrile to touch (Temp = 38°c). He had yellowish stain on nails (probably from meconium) and pale bluish extremities and no jaundice. Chest exam revealed thoraco-abdominal asynchrony, tachycardia (160b/min), no murmur and crackles in both lungs. Abdomen was apparently normal and anal canal was patent. A working diagnosis of Early onset neonatal sepsis following meconium aspiration syndrome (MAS) was made. Due to financial constraints only a Full Blood Count (which showed leukocytosis) and Chest X-Ray (which showed hyperinflation of the lung with patchy infiltrates in lower lung fields) could be afforded. Arterial blood gases were not done. Suctioning of oropharynx was done and the child placed on oxygen, kept warm and intravenous hydration and antibiotic therapy started. After 2 days of treatment, there was improvement in general condition. Day 4 of admission was marked by choking and regurgitation following commencement of feeds but no fever. A repeat FBC was normal. Mother was re-assured and treatment continued. Day 5 was marked by persistence of regurgitation, difficulty breathing during feeds with bluing of periphery and frothy salivation for which mother reports to have been present since birth. No abdominal distension and the child passed stool. The differential diagnosis now included; Oesophageal atresia, oesophageal web, oesophageal stricture, respiratory failure. Nasogastric tube inserted which coiled returning towards oral cavity and a repeat Chest and Abdominal X-ray with tube in place showed mild pulmonary infiltrates with no air in the stomach and duodenum respectively. This was suggestive of an oesophageal atresia with proximal trachea-oesophageal fistula. The child was re-examined thoroughly and no evidence for other congenital anomaly was seen. A good samaritan offered for the Abdominal and Cardiac echography which were normal. The neonate was then referred to Paediatric surgeon at a referral centre for management. Due to low birth weight and inadequate facilities, child was managed conservatively by parenteral nutrition, antibiotics and upper pouch suction pending eventual surgical correction when considered to be low risk. We lost the child 16 hours after arrival at referral centre.

## Discussion

The year 1697 saw the first description of Oesophageal atresia and Tracheo-oesophageal fistual by Thomas Gibson. Oesophageal atresia (OA) is a congenital malformation in which the oesophageal lumen is found to be interrupted, resulting in an upper and lower segment. While a vast majority of patients (92%) usually have a trachea-oesophageal fistula (TOF), about 4% of patients with TOF do not have OA [4]. This occurs in 1/2500 – 4500 live births [4–6]. The aetiology of oesophageal atresia is poorly understood. The trachea, oesophagus and lung are foregut derivatives, and during the fourth week of embryonic life, the foregut divides into ventral respiratory and dorsal oesophageal components. It is suggested that there is an alteration in the migration of the lateral folds or growth arrest at the time of evagination. Most of the time, the posterior oesophagus does not completely separate from the trachea, leading to distinct varieties of trachea-oesophageal fistula (TOF). This occurs between the third and sixth week of gestation [7]. The birth of an infant with OA/TOF in a family without a previous history of this condition is associated with a recurrence risk of ~ 1%. Twin concordance rate for OA/TOF is about 2.5% [4]. The above information suggests that genetic factors play a minor role in the pathogenesis of OA/TOF, though chromosomal anomalies like trisomy 18 and 21 predispose to this condition. Even more recently, three genes associated with OA/TOF in humans have been identified [4].

Anatomically, there are five subtypes (Gross & Vogt classification) of Oesophageal atresia and this is based on their relative frequencies; Type A: OA with distal TOF (86%), Type B: Isolated OA (8%), Type C: Isolated TOF (4%), Type D: OA with proximal TOF (1%), Type E: OA with double TOF (1%). Our case constitutes a rare subtype whose relative frequency is ~1% [4, 5]. It should however be noted, that in cases of isolated OA which is slightly more common than OA with proximal TOF, a similar presentation could also be found, thus, proper surgical exploration or autopsy would however be necessary for a definitive confirmation of the latter. Our diagnosis was however suggestive, due to the suctioning of maternal milk from the trachea and lung infiltrates found on chest x-ray.

50% of newborns with OA/TOF are said to have associated anomalies (Table 1) [4] with congenital heart disease being the commonest with a major determinant for survival.

**Table 1:** **Congenital anomalies associated with OA and TOF (Liverpool series 1953–97)**

Type	581 cases n(%)
Cardiac	154 (27%)
Urogenital	105 (18%)
Skeletal	71 (12%)
Vertebral	64 (11%)
Anorectal	67 (12%)
Gastrointestinal	53 (9%)
Palatal/laryngotracheal	44 (8%)
VACTERL	25 (19%)

Several phenotypic variants have been identified; the VACTERL association (vertebral, anorectal, cardiac, trachea, esophageal, renal and limb abnormalities) has been described. Though rare, a number of syndromes have also been associated like Holt-Oram syndrome, the DiGeorge syndrome, polysplenia and the Pierre-Robin syndrome [4].

The diagnosis of OA/TOF can be made in the antenatal period with the help of an Ultrasound scan which will show polyhydramnios and the proximal dilated blind ending oesophageal pouch [8, 9]. It should be noted that features in the prenatal period could be missed and many other conditions could also present with polyhydramnious and small or absent air bubble in the stomach. Generally if suspected, this gives room for closer follow-up of pregnancy and delivery in specialist centre thus preventing inadvertent feeding and pulmonary aspiration pneumonitis. In our case, this was missed as the mother had no ultrasound scan prenatally due to financial constraints. In the post natal period, OA and TOF should be suspected if a newborn is noted to have difficulty in clearing saliva, repeated episodes of coughing and choking (especially following feeds, as was the case in our patient), or transient cyanosis shortly after birth. Inability to pass a rigid nasogastric tube down the oesophagus can confirm the suspicion with a plain abdominal X-ray showing the chest to demonstrate the coiled tube in the oesophagus being more confirmatory. Additionally, the presence of air in the stomach and intestine is suggestive of the presence of TOF and its absence rules it out(isolated OA) and a dilated upper pouch is more suggestive of TOF. Upper pouch TOF occurs in less than 1% of cases and could easily be missed immediately after birth. In the presence of upper pouch esophagogram (UPEG) and tracheobronchoscopy, the diagnosis could be made. Thus recently, contrast Oesophagogram with fluoroscopic control and even endoscopic procedures like bronchoscopy and oesophagoscopy are being used, though the former must be done by an experienced radiologist and in a setting with adequate emergency neonatal resuscitation facilities due to the risk of aspiration pneumonia & lung injury from the contrast [10]. Barium offers best visualization as contrast but extraluminal barium can cause fibrous and granulomatous reactions leading to fibrous mediastinitis. Aqueous low osmolality agents like Optiray and Visipaque are preferred for use as they have less deleterious effects on the digestive system though are more expensive. These aqueous products are generally preferred in neonates and preterm especially with oesophageal perforation as they stay for long periods in the gut and are not easily absorbed. The shortcoming with aqueous contrast is their decrease coating ability leading to less fluoroscopic visibility. Hyperosmolar agents are usually contraindicated as they could cause irritation and pulmonary oedema if aspirated. In diagnosis of the rare proximal fistuala, barium swallow may fail to demonstrate this anomaly but videofluoroscopic studies during cautious filling of the proximal pouch would visualize it [11]. Magnetic Resonance Imaging has very little role in diagnosis of EA and TOF but 3D CT scan has 100% sensitivity and specificity for oesophageal atresia and hence most reliable [12]. The diagnosis of this condition in developed world rarely exceeds 20 hours [13] as opposed to 4.4 days in our low-income setting [14] Another shortcoming for diagnosis in our case was the absence of these diagnostic modalities. Due to the vast range of associations, it is important to investigate for other anomalies especially cardiac, as their treatment may take priority over correction of OA/TOF. In our case, no other congenital anomaly was identified.

Surgical correction is adviced to be urgent as delay increases the risk of aspiration of saliva from the upper pouch or reflux of gastric acid through the lower pouch and a TOF causing pneumonitis. Cardiac ultrasonography is of prime importance to demonstrate presence of cardiovascular anomaly that could affect anaesthetic management or surgical approach as thoracotomy is usually performed opposite to the side of the aortic arch [15]. Surgical management would involve a setting with an upto date Neonatal Intensive Care Unit and appropriate anaesthesia. These are readily available in the developed world giving current survival rates of almost 100%. This is not the case in low-income settings like ours, without all the above facilities, as morbidity and mortality rates are still high with some patients even dying before surgery [14].

The complications in the post-operative period are vast, including structural and functional problems. In the early post-op period, they range from tracheomalacia, repeated chest infections, anastomotic leak (11-21% of patients) and upto 50% developing oesophageal stricture, and pneumothorax from disruption. Gastro-oesophageal reflux (GOR) occurs in 35-58% of patients [16]. Late complications range from respiratory (46%) with 19% being recurrent pneumonia and 23% having repeated episodes of aspiration. It should be noted that these respiratory complications are secondary to GOR (74%), tracheomalacia (13%), recurrent TOF (13%), or oesophageal stricture (10%). Generally, recurrence of TOF occurs in about 9% of cases, typically 2-12 months after surgery and is more likely if there was excessive mobilization of the oesophagus during surgery, anastomotic leak and oesophageal stenosis [17]. The outcome for this condition can be seen in the Spitz classification system (Table 2) [18] based on birth weight and presence or absence of major congenital heart disease.

**Table 2:** **The spitz classification**

Group	Features	Survival (%)
**I**	Birth weight >1500 g, no major cardiac anomaly	98.5
**II**	Birth weight <1500 g, or major cardiac anomaly	82
**III**	Birth weight <1500 g and major cardiac anomaly	50

Generally, the mortality rate for OA/TOF remains on the decline in the developed world currently <1.5% for patients without major cardiac anomalies and with birth weight of >1500 g. The outcome is generally better for term babies than preterm. The fatality in the case reported was most probably due to delay in diagnosis, prematurity and inadequate management modalities.

## Conclusion

This illustrates the importance of detailed clinical examination in newborns. Thorough prenatal work-up is also important as the Oesophageal atresia may have been diagnosed earlier by ultrasound scan. Oesophageal atresia with proximal TOF is rare, and its rarer association with MAS together with absence of up to date diagnostic and management facilities, most likely led to delay in diagnosis in our case. A high index of suspicion for TOF should exist when faced with a newborn with the triad: respiratory distress especially during feeds, regurgitation and persistent frothy salivation.

## Consent

Written informed consent was obtained from the parents of the child for publication of this case report. A copy of the written consent is available for review by the Editor-in-Chief of this journal.
